# Metabolic Reprogramming in Triple-Negative Breast Cancer

**DOI:** 10.3389/fonc.2020.00428

**Published:** 2020-03-31

**Authors:** Xiangyu Sun, Mozhi Wang, Mengshen Wang, Xueting Yu, Jingyi Guo, Tie Sun, Xinyan Li, Litong Yao, Haoran Dong, Yingying Xu

**Affiliations:** Department of Breast Surgery, The First Affiliated Hospital of China Medical University, Shenyang, China

**Keywords:** triple negative breast neoplasms, metabolic networks and pathways, neoplasm metastasis, tumor microenvironment, glycolysis

## Abstract

Metabolic reprogramming is an emerging hallmark of cancer cells, in which cancer cells exhibit distinct metabolic phenotypes to fuel their proliferation and progression. The significant advancements made in the area of metabolic reprogramming make possible new strategies for overcoming malignant cancer, including triple-negative breast cancer. Triple-negative breast cancer (TNBC) is associated with high histologic grade, aggressive phenotype, and poor prognosis. Even though triple-negative breast cancer patients benefit from standard chemotherapy, they still face high recurrence rates and are more likely to develop resistance to chemotherapeutic drugs. Therefore, there is an urgent need to explore vulnerabilities of triple-negative breast cancer and develop novel therapeutic drugs to improve clinical outcomes for triple-negative breast cancer patients. Metabolic reprogramming may provide promising therapeutic targets for the treatment of triple-negative breast cancer. In this paper, we primarily discuss how triple-negative breast cancer cells reprogram their metabolic phenotype and that of stromal cells in the microenvironment to survive under nutrient-poor conditions. Considering that metastasis and chemoresistance are the main contributors to mortality in triple-negative breast cancer patients, we also focus on the role of metabolic adaption in mediating metastasis and chemoresistance of triple-negative breast cancer tumors.

## Introduction

Triple-negative breast cancer (TNBC) is a heterogeneous group of breast cancers, characterized by lack of expression of estrogen receptors, progesterone receptors and human epidermal growth factor receptor 2 gene amplification, making it unresponsive to endocrine therapy and HER2-targeted treatment. TNBC accounts for nearly 15% of all invasive breast cancers, and it has the highest rate of metastatic occurrence and poorest overall survival of all subtypes of breast tumors (1, 2). Due to the absence of approved targeted therapy, cytotoxic chemotherapy is the current primary established systemic therapy for early and advanced TNBC disease (3). Although chemotherapy significantly improves clinical outcomes for TNBC patients, recurrence rates remain relatively high and TNBC tumors often develop resistance to chemotherapeutic agents (3, 4). Thus, considering the limited treatment options and aggressive phenotypes of TNBC, it is crucial to improve our understanding of TNBC features and discover potential therapeutic targets to aid in the development of effective therapies.

A general characteristic of cancer cells is the capability to obtain nutrients from a nutrient-deprived environment and to use these nutrients to sustain their transformed state, build biomass, and increase cell proliferation (5–7). The major metabolic reprogramming occurring in tumors was first identified by Otto Warburg. He discovered that cancer cells maintain high levels of glycolysis for ATP production, regardless of oxygen availability, a phenomenon termed as the Warburg effect (8). From then on, studies began to focus on cancer-associated metabolic reprogramming within crucial metabolic pathways, including altered metabolism of glucose, lipids, and amino acids, to explore potential metabolic vulnerabilities during cancer progression.

In this review, we first introduce the major molecular features of TNBC related to metabolic reprogramming. In addition, we sum up the potential metabolic targets and corresponding agents for TNBC treatment in preclinical/clinical phases (Table 1) and give an overview of the major metabolically adapted pathways in TNBC tumors, primarily glucose, fatty acid, and amino acid metabolism, then explore potential therapeutic targets for metabolic vulnerabilities to guide TNBC therapy. We further summarize the metabolic interaction between TNBC tumors and microenvironment and how metabolic adaption in TNBC tumors influences the metastatic process and chemoresistance.

**Table 1:** Potential metabolic targets of triple-negative breast cancer.

**Target**	**Drug**	**Metabolic effect**	**Phase**	**References**
**GLUCOSE METABOLISM**				
GLUT	Metformin	Blocking glucose uptake	Preclinical	(9)
Hexokinase2	2-DG	Blocking aerobic glycolysis	Preclinical	(10, 11)
LDHA	Galloflavin, metformin	Blocking aerobic glycolysis	Preclinical	(9, 12)
Mitochondrial protein translation	Tigecycline	Blocking mitochondrial protein translation	Preclinical	(13)
Mitochondrial complex I	AG311	Blocking mitochondrial oxidative metabolism	Preclinical	(14)
**FATTY ACID METABOLISM**				
CPT1	etomoxir	Blocking fatty acid oxidation	Preclinical	(15)
FASN	EGCG	Blocking fatty acid synthesis	Clinical phase1	(16, 17)
**GLUTAMINE METABOLISM**				
SLC1A5	V-9302	Inhibiting glutamine uptake	Preclinical	(18)
xCT	SASP	Blocking cystine/glutamate exchange	Preclinical	(19)
GLS	CB839	Blocking conversion of glutamine to glutamate	Clinical phase1/2	(20)
AMINOTRANSFERASE	AOA	Blocking conversion of glutamate to α-KG	Preclinical	(21)
**SERINE METABOLISM**				
PHGDH	CBR-5884	Blocking serine biosynthesis	Preclinical	(22)

## Molecular Features of TNBC Related to Metabolic Reprogramming

Characterization of the molecular features of TNBC is essential for identifying the events that drive tumorigenesis. These tumorigenic effects can be accomplished by affecting the metabolic reprogramming in TNBC cells. MYC amplification is a crucial molecular event in TNBC cells (23). The MYC oncogene is overexpressed in 40% of TNBC and it encodes a transcription factor, c-myc, that links metabolic reprogramming to tumorigenesis (23, 24). C-myc interacts with another helix–loop–helix leucine zipper protein, MYC associated factor X (Max), to bind specific DNA sequences and regulate gene expression for its transcriptional activity (25). C-myc can not only directly transactivate genes involved in metabolic pathways but also cooperate with other crucial metabolic drivers, such as hypoxia inducible factor 1-alpha (HIF-1α), to facilitate critical cellular processes for survival (26, 27). Thus, MYC amplification is crucial for the metabolic rewiring of TNBC cells to promote the tumorigenesis. p53 is the most-frequently mutated gene in TNBC, with a frequency up to 80% (23). Tumor suppressor p53 plays a crucial role in maintaining genomic stability in response to metabolic stress signals (28). Nevertheless, p53 mutations produce a protein with an impaired ability to bind to specific DNA sequences, leading to dysregulated p53 transcriptional pathway (29). p53 is activated by phosphorylation of AMP-activated protein kinase (AMPK). Upon activation, p53 induces a response of reversible cell-cycle checkpoint under nutrient-deprived conditions (30). However, deficiency of this response in cancer cells with p53 mutations may enhance the proliferative capability during nutrient limitation. Recently, loss of Beclin-1 has also been characterized as another key molecular alteration emerged in TNBC cells (31). Beclin-1 (BECN1) has been identified as a tumor suppressor that participates in the lysosomal degradation pathway to enhance autophagy and it is expressed at lower levels in mammary carcinomas, particularly TNBC (31–33). A key mechanism of autophagy is to scavenge broken organelles such as mitochondria and unfolded proteins via autophagy-related proteins. Besides, autophagy-related proteins can also regulate metabolic reprogramming through modulation of key metabolic enzymes. For example, impaired autophagic activity leads to enhancement of glycolysis to support the survival of cancer cells (34). Thus, the autophagy-related BECN1 may be a negative regulator of metabolic rewiring that favors the mammary carcinogenesis. Consistently, loss of BECN1 and autophagy may serve as a link between metabolic reprogramming and carcinogenesis in TNBC. Compared to other subtypes of breast cancer, TNBC also exhibits molecular features including increased loss of PTEN, lower PIK3CA mutation and RB1 expression, which also have great significance for endowing a distinct metabolic phenotype of TNBC cells for their survival under metabolic stress conditions (23).

## The Metabolic Phenotype of TNBC

### Glucose Metabolism

**Figure 1:**
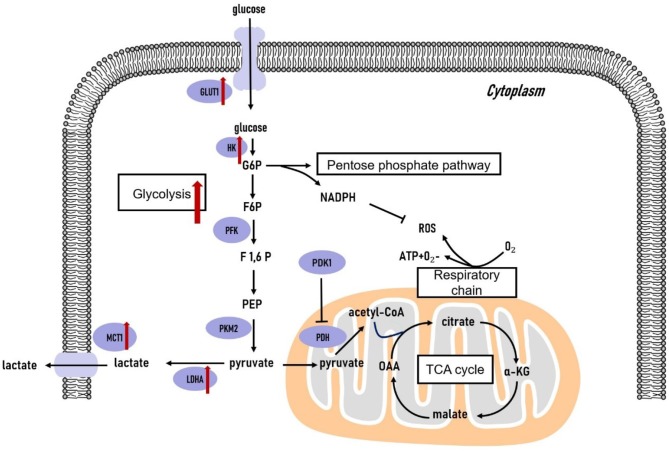
Glucose metabolism in triple-negative breast cancer cells. The glycolytic pathway is significantly upregulated in triple-negative breast tumors. The genes coding the key glycolytic enzymes are overexpressed in triple-negative breast tumors. TCA, tricarboxylic acid cycle; G6P, glucose-6-phosphate; F6P, fructose-6-phosphate; F1,6P, fructose-1,6-bisphosphate; PEP, phosphoenolpyruvate; OAA, oxaloacetate; α-KG, α-ketoglutarate; GLUT, glucose transporter; HK, hexokinase; PFK, phosphofructokinase; PKM2, pyruvate kinase isozyme type 2; LDHA, lactate dehydrogenase A; MCT1, monocarboxylate transporter 1; PDK1, pyruvate dehydrogenase kinase 1; PDH, pyruvate dehydrogenase.

Cancer cells transform their glucose metabolic phenotype to adapt to the accelerated rate of proliferation, invasion, and migration. The shifted glucose metabolism (illustrated in Figure 1) in cancer cells satisfies their energy demand through aerobic glycolysis in the cytoplasm rather than relying on mitochondrial oxidative phosphorylation (OXPHOS), as is the preferential mode of energy generation in normal cells (35). Glycolysis is generally separated from OXPHOS in cancer cells. As a result, glycolysis-derived pyruvate is primarily diverted to lactate fermentation rather than to OXPHOS. The remodeled metabolic phenotype not only allows rapid energy generation in terms of ATP but also provides sufficient glycolytic intermediates to support anabolic demands in cancer cells (36, 37). In this section, we primarily focus on the metabolic phenotype of glycolytic dependence and altered mitochondrial oxidative metabolic activity in the glucose metabolism pathway to provide a better understanding of their role in supporting TNBC tumor cells.

#### Glycolysis

Intensive glucose uptake is a crucial trait of TNBC (38, 39). Glucose transporter 1 (GLUT1) is an essential protein in the glucose metabolism pathway that provides cells with glucose by constantly fostering the transportation of glucose across the plasma membrane (40). GLUT1 overexpression is significantly correlated with TNBC, and it promotes TNBC cell proliferation and invasion (38, 39). Notably, serine/threonine kinase AKT stabilizes GLUT1 at the cell membrane by perturbing its endocytosis, therefore promoting aerobic glycolysis (41). USP6NL, a GTPase-activating protein involved in signal transduction regulation, is often overexpressed in basal-like TNBC. Knockdown of USP6NL impairs epidermal growth factor receptor (EGFR)/AKT signaling and promotes GLUT1 degradation, thus suspending cell proliferation exclusively in aggressive basal-like TNBC tumors harboring USP6NL overexpression (41).

Once transported into cells, glucose undergoes glycolysis to produce pyruvate. In cancer cells, pyruvate is continually converted to lactate in the cytoplasm, thus hampering glucose entry into mitochondria. Glycolysis-related enzymes are highly expressed in TNBC tumors, implying high glycolytic activity in their development (36, 42). Hexokinase 2 (HK2) is the most extensively studied isoform of Hexokinase (43). MiR-155 binds to transcriptional promoter STAT3 to facilitate HK2 transcription and it also acts to repress a negative regulator of HK2, miR-143, leading to elevated HK2 expression at the post-transcriptional level and enhanced glycolytic phenotype of TNBC. Thus, the miR-155, miR-143, and HK2 axis may provide potential therapeutic targets for TNBC (44). Indeed, systemic administration of chemically synthesized miR-143 mimic to TNBC xenografts demonstrates a significant reduction of both tumor growth and 18F-FDG uptake via PET/CT, confirming that miR-143 is an effective inhibitor of glycolysis and a promising therapeutic target for TNBC treatment (45).

The pyruvate kinase 2 (PKM2) isoenzyme is also crucial for the glycolytic phenotype and tumor growth of TNBC (46). PKM2 is highly expressed in TNBC cell lines and tissues compared to non-tumorigenic breast cancer cell lines and surrounding healthy mammary tissues (47). PKM2 knockdown has anticancer effects against TNBC cells, since it suppresses NF-kB activity by diminishing expression of phosphorylated p65 protein and dampening NF-kB target gene expression (47). Post-translational modifications of PKM2 are well investigated. PKM2 is regulated by various post-translational modifications through multiple oncogenic tyrosine kinases, which are inactivated in normal tissues but active in tumor tissues (48–50). For instance, PKM2 is phosphorylated at tyrosine 105 (Y105) and subsequently transformed to be oncogenic. Phosphorylation of PKM2-Y105 facilitates Yes-associated protein (YAP) nuclear translocation and therefore promotes cancer stem-like cell properties. Inversely, suppression of PKM2-Y105 phosphorylation significantly diminishes YAP nuclear translocation and cancer stem-like cells, leading to the retarded TNBC tumor growth (49). Thus, targeting PKM2 and its corresponding oncogenic post-translational modifications may be a promising clue for TNBC treatments.

Lactate dehydrogenase A (LDHA) catalyzes the step of aerobic glycolysis converting pyruvate to lactate in the cytoplasm. Compared to luminal breast cancer cells, TNBC cells exhibit higher LDHA levels and lower oxygen consumption rates (51). MiR-34a dampens the function of both programmed cell death receptor ligand 1 (PDL1) and LDHA. Moreover, PDL1 and LDHA act as rival endogenous RNAs by competing for miR-34a, identifying the crucial role of miR-34a as a tumor suppressor that modulate immunity and glycolysis in TNBC cells (52). Thus, LDHA inhibitors may serve as beneficial drugs for attenuating the TNBC development (10, 53). Monocarboxylate transporters (MCTs), particularly MCT1, serve as the lactate transporters in cancer cells. Malignant breast cancer subtypes, such as TNBC, demonstrate preferentially high expression of MCT1 (54). Silencing MCT1 in basal-like TNBC cells impairs lactate efflux, cell proliferation, and migration *in vitro* as well as tumor growth and formation *in vivo* (55). MCT1 is also a direct target of miR-342-3p, and loss of this miRNA increases the MCT1 expression, leading to enhanced glycolytic profile and more aggressive phenotype of TNBC tumor cells (56). Thus, loss of miR-342-3p and overexpression of MCT1 may indicate poor prognosis in TNBC patients. These findings have helped to drive the development of novel drugs targeting glycolytic enzymes for treating TNBC tumors.

The regulatory effects of critical transcriptional factors and oncogenic signaling pathways on the glycolytic phenotype of TNBC tumors are also under investigation. HIF-1α is a crucial regulatory factor of glycolysis in cancer cells under hypoxic conditions. HIF-1α is regulated by Nuclear factor erythroid 2-like-2 (NRF2), an essential regulator of multiple genes involved in overcoming oxidative stress (57). In TNBC cells, NRF2-silencing can suppress HIF-1α enrichment and sequentially lower expression of glycolysis enzymes. Specifically, dysregulated HIF-1α signaling in NRF2-silenced TNBC cells is induced by miR-181c, indicating that NRF2 and miR-181c may be novel targets for blocking HIF-1α-mediated glycolytic adaption in TNBC cells (58). C-myc is another oncogenic transcriptional factor regulating the glycolytic phenotype of TNBC tumors. C-myc can drive glycolytic programming by repressing thioredoxin-interacting protein (TXNIP), a key negative regulator of glucose uptake and aerobic glycolysis, exclusively in TNBC tumors. Interestingly, glucose uptake is attenuated in myc-knockdown TNBC cells, whereas glucose uptake recovers to the control group level in TNBC cells containing both TXNIP- and myc-knockdown. Moreover, the expression level of TXNIP and myc can predict clinical outcomes of TNBC patients. The TXNIP low/myc high gene signature only associates with decreased metastasis-free and overall survival in TNBC but not in other subtypes of breast cancer (42). Epidermal growth factor (EGF) signaling, which is highly activated in TNBC tumors, can also promote glycolysis of TNBC cells. EGF signaling upregulates HK2 expression and directly phosphorylates PKM2 at Y418 to impair its activity. These effects lead to accumulation of glycolytic intermediates, thus providing proliferative advantages for TNBC tumors. For instance, one metabolites, lactate, allows TNBC cells to evade destruction via cytotoxic T cells. A combination of an EGFR tyrosine kinase inhibitor, gefitinib, and a glycolysis inhibitor, 2-DG, is effective to block growth and progression of TNBC tumors (10). These key transcriptional factors and signaling pathways are indispensable for discovering interventions for TNBC tumors.

#### Mitochondrial Oxidative Metabolism

According to the Warburg effect, cancer cells experience a shift from OXPHOS to glycolysis under hypoxia and nutrient-deprived conditions. Nevertheless, both elevated and reduced OXPHOS activity is observed in TNBC cells. Lower OXPHOS activity may result from mitochondrial DNA (mtDNA) mutation or less mtDNA content coding for the subunits of OXPHOS protein complexes I to V (59). Compared to other subtypes of breast cancer, TNBC tumors display a higher frequency of mitochondrial defects (60). Thus, lower mtDNA content and respiration level are crucial characteristics of TNBC tumors, providing a clue for future precision therapy. In the context of increased OXPHOS activity, one current proposal is that cancer cells may simultaneously sustain glycolysis and OXPHOS at high levels. For instance, OXPHOS is highly upregulated in TNBC with RB1 deficiency. RB1 forms a complex with E2F to bind promoters of mitochondrial protein translation (MPT) genes to regulate their transcription. Specifically, MPT genes are induced by E2F1 and suppressed by RB1. Thus, RB1 deficiency has a prototypic oncogenic property to facilitate mitochondrial OXPHOS. These findings reveal that either RB1 loss or induction of E2F1 facilitates TNBC cells proliferation by affecting MPT genes on a transcriptional level. Thus, the FDA approved MPT antagonist tigecycline (TIG) exhibits a considerable inhibitory effect on the proliferation of RB1-deficient TNBC cells, and it is a clinically approved drug for RB1-deficient TNBC (13, 61). It has been proposed that changes in some oncogenes or tumor suppressors, including RB1 deficiency, may enhance OXPHOS. Therefore, it is crucial to identify these oncogenic alterations to determine the mitochondrial oxidative metabolic activity in TNBC tumors, which may aid in TNBC treatments.

### Fatty Acid Metabolism

**Figure 2:**
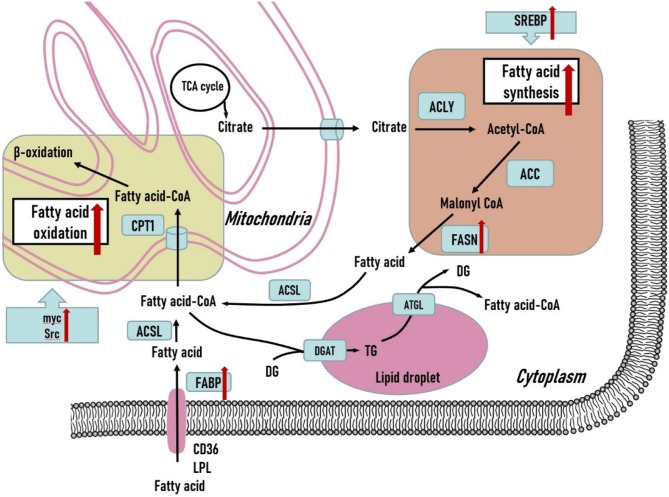
Fatty acid metabolism in triple-negative breast cancer cells. Both fatty acid synthesis and fatty acid oxidation are upregulated to support the development of triple-negative breast tumors. Various crucial genes related to fatty acid metabolism are overexpressed in triple-negative breast tumors. Additionally, several oncogenic genes are also upregulated to promote fatty acid oxidation in triple-negative breast tumors and may serve as potential therapeutic treatments. DG, diglyceride; TG, triglyceride; ACLY, ATP citrate lyase; ACC, acetyl-CoA carboxylase; FASN, fatty acid synthase; ACSL, long-chain acyl-coenzyme A synthase; CPT1, carnitine palmitoyl transferase 1; DGAT, diacylglycerol acyltransferase; ATGL, adipose triglyceride lipase; LPL, lipoprotein lipase; FABP, fatty acid binding protein.

Aside from the crucial role of glucose metabolism in TNBC tumors, fatty acid metabolism, including fatty acid synthesis and fatty acid oxidation, is an important part of the metabolic phenotype of TNBC tumors. Fatty acid synthesis and fatty acid oxidation are generally viewed as counterparts in metabolic reprogramming of tumor cells. However, both of the metabolic pathways play essential roles in supporting the TNBC progression. The fatty acid metabolism is illustrated in Figure 2.

#### Fatty Acid Synthesis

Normal fatty acid synthesis primarily occurs in lipogenic tissues, especially adipose tissue (62). Nevertheless, fatty acid synthesis is enhanced by oncogenic signaling in tumor cells for the production of membrane phospholipids and signal molecules to prepare for tumor cell proliferation. Unlike normal mammary cells which mainly depend on extracellular lipids for building biomass, tumor cells enhance the *de novo* fatty acid synthesis to satisfy their needs for energy and intermediates under conditions of metabolic stress (62).

Fatty acid synthase (FASN) has been found to be overexpressed in TNBC tumor cells (63). In preclinical models of TNBC tumors, FASN inhibitors in combination with anti-EGFR signaling agents show significant anti-tumor effects in TNBC tumors (64). Sterol regulatory element-binding protein (SREBP) is the master transcriptional regulator of lipogenic enzymes including ATP-citrate lyase, acetyl-CoA carboxylase, and FASN. SREBP can promote *de novo* fatty acids synthesis via transcriptional activation of corresponding lipogenic genes (65). O-GlcNAc transferase also regulates SREBP-1 phosphorylation in an AMPK-dependent manner and targets the transcription of key lipogenic enzymes to regulate lipid metabolism and growth of breast cancer cells. O-GlcNAc transferase inhibition slows TNBC tumor growth *in vivo*, and SREBP-1 overexpression can partially rescue this blockage (66). These findings imply a crucial role of SREBP in facilitating the lipogenic process to support malignant behaviors in TNBC tumor cells.

#### Fatty Acid Oxidation

Fatty acid oxidation (FAO) is a multi-step process that allows long-chain fatty acids to convert into fatty acid-CoA and enter the tricarboxylic acid (TCA) cycle and OXPHOS to be completely oxidized to sustain ATP production (67). FAO serves as an indispensable source of ATP, NADH, and NADPH, providing survival advantages to fuel growth of TNBC tumor cells (68). TNBC tumor cells rely on FAO to support proliferation, invasion, and metastasis. Intriguingly, FAO regulates proliferation and metastasis in TNBC tumors in a Src-dependent manner. Carnitine palmitoyltransferase (CPT) is capable of importing long-chain acyl-CoAs across the mitochondrial inner membrane into the mitochondria (69). The expression level of CPT1A positively correlates with Src phosphorylation status, which is only found in basal-like TNBC subtypes. Src is located in the mitochondria and it is responsible for phosphorylating multiple proteins related to the mitochondrial electron transfer chain (ETC). FAO promotes autophosphorylation of the Src oncoprotein at Y419 in TNBC cells, and the activated Src can further phosphorylate mitochondrial ETC proteins to sustain mitochondrial function to produce sufficient energy for TNBC cells metastasis (70). Therefore, FAO-driven Src plays a key role in endowing TNBC tumor cells with oncogenic capability. FAO is also of great significance in MYC-overexpressing TNBC tumor cells, in which FAO pathway is significantly upregulated. Small molecule inhibition and knockdown of CPT demonstrate remarkable inhibitory effects in MYC-overexpressing TNBC cells compared to TNBC cells with a MYC-low signature. It is feasible that TNBC is responsive to FAO inhibition in an MYC-dependent mode (71).

#### Fatty Acid Uptake and Storage

In addition to *de novo* fatty acid synthesis, TNBC cells can also obtain fatty acids through absorbing dietary lipids from the blood circulation or by uptake of exogenous fatty acids derived from adipocytes in the microenvironment. Both require a series of fatty acid transporters. In extracellular lipolysis, lipoprotein lipase (LPL) is highly expressed in TNBC (72). LPL is a secreted enzyme that catalyzes hydrolysis of circulating triglycerides in chylomicrons or very low-density lipoproteins to produce free fatty acids. The fatty acids generated from TGs hydrolysis can then be taken up by TNBC cells via CD36 (73). Thus, TNBC cells demand both *de novo* fatty acid synthesis and LPL-mediated extracellular lipolysis followed by fatty acid uptake via CD36. Fatty acid-binding proteins (FABPs) are a series of cytoplasmic proteins that facilitate fatty acid entry into cells. FABP5 is associated with poor survival rates in TNBC patients (74). Moreover, loss of FABP5 in TNBC tumor cells inhibits proliferation and invasion *in vivo* (74, 75). FABP5 is essential for EGFR expression, and loss of FABP5 contributes to the proteasomal degradation of EGFR. Thus, FABP5 is required for the EGF-mediated metastatic process (75). FABP5 also facilitates the generation of fatty acids through lipolysis of lipid droplets (LDs) and *de novo* fatty acid synthesis to promote TNBC tumor progression (76). FABP7 is generally expressed in normal mammary cells, and FABP7 overexpression emerges in TNBC (77). Under serum deprivation, FABP7-overexpressing TNBC cells experience S/G2 phase arrest and cell death via peroxisome proliferator-activated receptors (PPAR-α)-mediated signaling. Interestingly, CPT1 is a target of PPAR-α-mediated signaling, and the fatty acids provided by LDs may prepare for the concomitant FAO (78).

LDs generally store neutral lipids in the form of triacylglycerol and cholesterol esters. Raman imaging and spectroscopy have been used to detect the accumulation of LDs in MCF10A, MCF7, MDA-MB-231 cell lines. Cytoplasmic LDs are considerably elevated in highly malignant MDA-MB-231 cells compared to moderately malignant MCF7 breast cells, with even lower levels in non-malignant MCF10A breast cells (79). These findings suggest that LD accumulation may correlate with increased breast cancer malignancy. The LDs accumulated in epithelial breast cancer cells differ in chemical composition compared to surrounding adipocytes, suggesting that LDs play different roles in epithelial breast cancer cells and adipocytes (79). The human group X secreted phospholipase A2 (hGX sPLA2), an enzyme that liberates fatty acids from lipoproteins, can induce LD formation inside TNBC cells to protect survival and proliferation in a serum-deprived environment (80). This effect of hGX sPLA2 may also correlate with activated AMPK pathway signaling to promote metabolic reprogramming in TNBC (81). Through induction of hGX sPLA2, LDs emerge as antioxidant and pro-survival hubs that protect TNBC cells from nutrient deprivation and lipotoxic stress by balancing unsaturated fatty acid sequestration and their liberation from LDs. Lipotoxicity derived from polyunsaturated fatty acids is eliminated when polyunsaturated fatty acids are sequestered in LDs, either via sPLA2-mediated triglyceride synthesis or suppression of adipose triglyceride lipase-induced triglyceride lipolysis (80). These findings indicate that suppressing triglyceride synthesis, facilitating LD formation, and promoting lipolysis may all be potential targets to cause TNBC cell death. Knockdown of FABP5 in TNBC cell lines induces accumulated cytoplasmic LDs with many clustered LDs transforming into dispersed LDs. This transformation is hypothesized to prepare for FAO (76). These studies indicate that LDs have multi-faceted roles in modulating TNBC metabolism, and further studies are required to lift the veil of LDs.

### Amino Acid Metabolism

**Figure 3:**
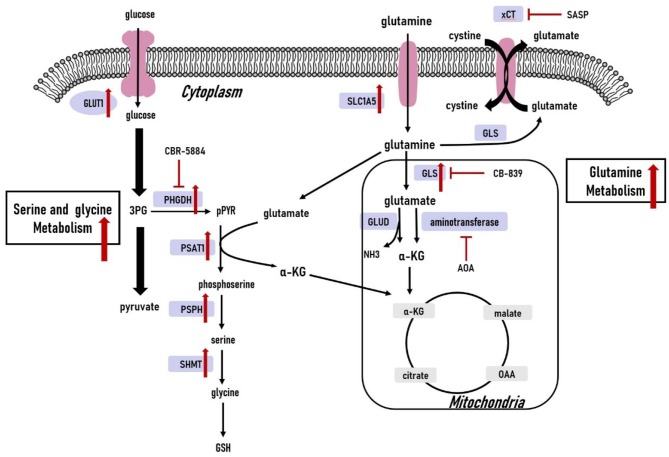
Amino acid metabolism in triple-negative breast cancer cells. The glutamine, serine and glycine metabolic pathways are significantly upregulated. The upregulated expression of key genes within the glutamine, serine and glycine metabolic pathways have also been observed in triple-negative breast cancer cells. 3PG, 3-phospho-glycerate; pPYR, phosphohydroxypyruvate; GSH, reduced glutathione; OAA, oxaloacetate; α-KG, α-ketoglutarate; GLU, glutamate; GLUT1, glucose transporter1; PHGDH, phosphoglycerate dehydrogenase; PSAT1, phosphoserine aminotransferase 1; PSPH, phosphoserine phosphatase; SHMT, serine hydroxymethyltransferase; SLC1A5, solute carrier family 1 member 5; xCT, cystine/glutamate antiporter; GLS, glutaminase; GLUD, glutamate dehydrogenase; ASNS, asparagine synthetase.

Cancer cells need enriched amino acids to sustain their survival in a nutrient-poor environment (82). Here, we discuss well-studied amino acids including glutamine, serine, and glycine to summarize the metabolic phenotype of TNBC tumors. Amino acid metabolism is illustrated in Figure 3.

#### Glutamine Metabolism

Glutamine is the most abundant amino acid in cancer cells. The significance of glutamine in cancer originates from its capability to contribute nitrogen and carbon to a series of reactions supporting cancer cell proliferation, invasiveness, and metastasis (82–84). First, glutamine provides carbon sources for entry into the TCA cycle by generating α-ketoglutarate (α-KG). Second, glutamine also provides nitrogen for nucleotide and non-essential amino acid biosynthesis. Third, glutamate generated from glutaminolysis is a precursor of glutathione, involved in maintaining redox balance (85). Glutamine uptake and glutamine-related enzymes are upregulated in TNBC cells (85, 86). Indeed, glutamine provides a survival advantage for TNBC tumor cells, therefore TNBC cells often exhibit glutamine-dependent phenotype (85, 86). Moreover, TNBC may be more susceptible to glutamine-targeting therapeutics compared to luminal types (85).

Glutamine metabolism begins with its solute carrier family 1 member 5 (SLC1A5)-dependent transport into cells (87). TNBC displays significantly increased SLC1A5-mediated glutamine uptake compared to luminal breast cancer cells (86). Additionally, inhibition of SLC1A5-mediated glutamine transport by knockdown of SLC1A5 or adding SLC1A5 inhibitor L-γ-glutamyl-p-nitroanilide (GPNA) attenuates mTOR activation and proliferation of breast cancer cells, and these inhibitory effects are more prominent in TNBC tumor cells compared to luminal subtypes (88). Furthermore, loss of SLC1A5 is sufficient to comprise growth of basal-like TNBC cells *in vitro* and *in vivo* (88). This subtype-specific effect of glutamine metabolism in TNBC tumors makes SLC1A5 a druggable metabolic target for TNBC treatment. V-9302, a promising small inhibitor specifically targeting SLC1A5, has been tested in preclinical models (18). Once transported into the cells, glutamine can either be exported by antiporters in exchange for other amino acids or participate in glutaminolysis to produce glutamate. The cystine/glutamate xCT antiporter is a dominant means of accumulating the intracellular cystine for glutathione synthesis, and it is expressed in approximately one-third of TNBC. Sulfasalazine (SASP) is an xCT inhibitor, which can significantly retard TNBC tumor growth *in vivo*, suggesting that xCT is a common druggable target in TNBC tumors (19). Glutamine is converted to glutamate through glutaminolysis catabolized by mitochondrial glutaminases (GLS). Studies have shown that GLS is necessary for TNBC tumor growth and survival. GLS1 loss in TNBC cell lines leads to dysregulated glutaminolysis, impairing tumor growth *in vitro* and *in vivo*, whereas GLS silencing has minimal effects on metabolic phenotype or growth in luminal cell lines. This may occur since basal-like, but not luminal, breast cancer cells are more glutamine-dependent because they lack expression of glutamine synthetase as an alternative to synthesize glutamine (86). Thus, TNBC cells may be more susceptible to GLS-targeting therapeutics. Glutaminase inhibitor CB-839, a potent and selective inhibitor of glutaminase, is currently in 1/2 clinical phase trails for malignant tumors, including TNBC. It has been found to be effective in attenuating the growth of TNBC and other glutamine-dependent tumors in xenograft models (20). Upon its production via glutaminase, glutamate is converted to α-KG through glutamate dehydrogenase or aminotransferases. Aminooxyacetate (AOA), an inhibitor of aminotransferases, shows anti-tumor effects in myc–overexpressing breast cancer through the endoplasmic reticulum stress response pathway in preclinical models. Since the majority of TNBC tumor cells harbor overexpression of myc and depend on glutamine for proliferation, AOA is a promising drug for TNBC treatment (21). The glutamine-dependent phenotype of TNBC tumors illustrate essential vulnerabilities of TNBC and may serve as effective treatments.

#### Serine and Glycine Metabolism

Serine and glycine are biosynthetically connected and produced from the glycolytic intermediate 3-phosphoglycerate (3PG). Serine is regarded as an inflection in the glucose metabolic pathway. Together, serine and glycine are the primary sources of one-carbon units used in the synthesis of nucleic acids, lipids, proteins, and cofactors (89). Diversion of 3PG out of glycolysis brings proliferative advantages for cancer cells. Various serine metabolism-associated enzymes, such as phosphoglycerate dehydrogenase (PHGDH), phosphoserine aminotransferase 1 (PSAT1), 1-3-phosphoserine phosphatase (PSPH), are highly expressed in TNBC tumors (90). Moreover, depletion of serine and glycine in culture media decreases TNBC cell proliferation (90).

3PG is oxidized into phosphohydroxypyruvate (pPYR) by PHGDH and approximately 9% of 3PG is diverted to the serine pathway. PHGDH overexpression has been observed in 70% of estrogen receptor-negative breast cancers, and high PHGDH expression indicates elevated serine synthetic flux of breast cancer cells (91). In PHGDH-overexpressing breast cancer cells, the synthetic serine pathway not only produces serine but also generate glutamate-derived α-KG. Nearly half of α-KG influx into the TCA cycle is from the synthetic serine pathway, which is crucial for proliferation of PHGDH-amplified breast cancer cells (91). This explains why the inhibitory effect of PHGDH suppression on breast cancer cell proliferation is unlikely to be rescued by the supplemented extracellular serine. Small molecule PHGDH inhibitors can abrogate the serine synthetic pathway and decrease proliferation of PHGDH-overexpressing breast cancer cells *in vitro* and *in vivo* (91, 92). Since the majority of TNBC tumors harbor PHGDH overexpression, PHGDH inhibition may be therapeutically valuable for treating of TNBC tumors. A PHGDH inhibitor, CBR-5884, has been found to dampen the proliferation of PHGDH-dependent TNBC tumor cells (22). Phosphoserine is produced from pPYR catalyzed by PSAT1, and it is then dephosphorylated by PSPH to generate serine. PSAT1 is up-regulated in estrogen receptor-negative breast cancer, and increased expression correlates with poor clinical outcomes (93). Unlike PHGDH, PSAT1 overexpression has not been found in TNBC tumor cells, and PSAT1 upregulation is generally presumed to result from responses to oncogenic signals. Elevated PSAT1 expression facilitates TNBC cell proliferation and invasion *in vitro* and *in vivo* by promoting cyclin D1 expression. In serine synthesis-independent TNBC cells, PSAT1 suppression blocks invasion without negatively affecting primary tumor growth. This may result from the fact that loss of PSAT1 affects F-actin cytoskeleton rearrangement and cell morphology in serine synthesis-independent TNBC cells, potentiating their metastatic ability (94). Since serine synthesis-independent TNBC cells are not responsive to PHGDH inhibitors, PSAT1 inhibition may provide a new solution for serine synthesis-independent TNBC cells. The serine metabolic pathway tends to be more essential for supporting TNBC proliferation and metastasis than glycine metabolism, making the serine metabolic pathway a primary candidate for TNBC treatment.

## Metabolic Interaction Between TNBC Cells and the Microenvironment

TNBC cells are sustained by adjacent stromal cells such as fibroblasts, macrophages and adipocytes. The interplay between TNBC cells and stromal cells contributes to the altered metabolic phenotypes in these cells, which may promote TNBC malignancy. In this section, we summarize the interplay between TNBC cells and their microenvironment.

### Metabolic Interaction With Tumor-Associated Macrophages

Tumor-associated macrophages (TAMs) are primary stromal cells that repress anti-neoplastic immunity and facilitate TNBC progression (95). Macrophages have generally been divided into M1 and M2 groups (96). TAMs normally possess functional properties of M2 macrophages (95, 97). Lactate serves as an essential metabolite for activating M2 macrophages (98–100). G protein-coupled receptor 132 (Gpr132) mediates the interplay between TAMs and breast cancer cells via sensing altered lactate to promote metastasis. When co-culturing TNBC cells with macrophages, acidic signals emerge to activate Gpr132-mediated M2 macrophages. Lactate is a key activation signal for Gpr132. Treating macrophages with IL-4, widely regarded as an M2 macrophage activator, demonstrates that Gpr132 serves as a sensor for lactate to activate lactate-induced M2 macrophages. The lactate-activated M2 macrophages consequently foster adhesion, migration and invasion of TNBC cells via Gpr132 *in vitro* and metastasis *in vivo* (100). TAMs also lead to intensified aerobic glycolysis and chemoresistance in TNBC cells via stabilizing HIF-1α. Indeed, this effect is realized by extracellular-vesicle transmitted HIF-1α-stabilizing long noncoding RNA (HISLA). Basal HISLA expression is determined by the transcription factor PU.1, while lactate serves as another positive regulator of HISLA expression. HISLA directly binds to prolyl hydroxylase domain 2 to interfere with the subsequent interaction between prolyl hydroxylase domain 2 and HIF-1α to prevent HIF-1α destabilization. Moreover, HISLA expression is correlated with the patient responsiveness to neoadjuvant chemotherapy, making HISLA a potential predictor for TNBC chemoresistance (101). These observations indicate that the tumor-M2 macrophage interaction as a novel mechanism that it may promote TNBC progression and that it should be a priority for the future research.

### Metabolic Interaction With Cancer-Associated Fibroblasts

Fibroblasts that have mutual effects on cancer cells are regarded as cancer-associated fibroblasts (CAFs). Several studies show CAF-conditioned medias increase TNBC cell proliferation (102–105). Interestingly, CAFs can recycle tumor-derived lactate as a source of energy when cocultured with TNBC cells, sparing glucose to be utilized by adjacent tumor cells, to reinforce the glucose supply to TNBC cells. CAFs also convert lactate to pyruvate, which can be further used for glycolysis and biosynthetic reactions in TNBC cells. Importantly, pyruvate also controls level of accumulated ROS in TNBC cells, supporting TNBC cell proliferation (102, 103). Coculture with TNBC cells causes CAFs to upregulate genes involved in glucose metabolism, especially glycolysis, while TNBC cells upregulate genes involved in the TCA cycle. Thus, interaction between TNBC cells and CAFs makes tumor cells more dependent on the TCA cycle, whereas CAFs are more reliant on glycolysis, consistent with CAFs providing TNBC cells with energy sources (105). Lipid transfer is also crucial in the metabolic junction between CAFs and cancer cells. In CAF-conditioned media, TNBC cells have higher lipid level, decreased FASN activity and increased fatty acid transport protein 1 (FATP1) transcriptional expression. Notably, CAFs display higher FASN activity and serve as hubs to supplement fatty acids for TNBC tumor cells. FATP1 mediates the fatty acid transport between TNBC cells and CAFs, making it a potential target to disrupt this lipid transfer to attenuate the TNBC tumor growth (104). The interplay between TNBC tumor cells and CAFs highlights potential vulnerabilities of TNBC tumors.

### Metabolic Interaction With Cancer-Associated Adipocytes

It is worth noticing that breast cancer tissues reside inside mammary glands surrounded by lipid-rich adipocytes. Peritumoral adipocytes that interact with tumor cells and exhibit altered metabolic phenotypes are termed as cancer-associated adipocytes (CAAs). Studies have illustrated that exposing TNBC cells to CAA-conditioned media or coculturing with adipocytes can alter the metabolic phenotype of TNBC tumor cells to enhance proliferation and invasion (106–108). Bidirectional crosstalk occurs between epithelial breast cancer cells and peritumoral adipocytes, including lipid transport. This crosstalk involves the ability of breast cancer cells to mediate the triglyceride lipase in surrounding CAAs to produce free fatty acids for energy storage and fuel supply. Coculturing experiments demonstrate that lipid transfer between TNBC cells and CAAs leads to increased FAO. This preferential uncoupling of FAO induced by peritumoral adipocytes is sufficient to drive TNBC cell invasion and metastasis (106, 107). Intriguingly, the rate of fatty acid transfer from CAAs was higher in TNBC cells than in luminal cells, indicating that the increased potential of fatty acid transfer potential may contribute to the high malignancy of TNBC. Furthermore, a range of paracrine signaling factors and altered gene expressions explain how CAAs alter the metabolic phenotype of breast cancer cells, including overexpressing proteases and elevating adipokines, including interleukin 6 (IL-6) (108). CAA-derived IL-6 plays a crucial role in the metabolic adaptation of TNBC tumor cells, leading to a more invasive phenotype. Adding murine IL-6 blocking antibody to cocultured 4T1 cells and adipocytes significantly attenuates the pro-invasive effect of CAAs, illustrating the role of IL-6 in mediating TNBC development. Their support to TNBC tumors implicate CAAs as potential targets for impeding TNBC tumor progression.

## Impact of Metabolic Reprogramming on TNBC Metastasis

Metastasis is the primary contributor to death for TNBC patients. The molecular mechanisms involved in the TNBC metastasis are being explored extensively, yet knowledge regarding the metabolic reprogramming of TNBC tumors during the metastatic process is desperately needed to find potential therapeutic targets.

Metastasis to sentinel lymph nodes (LN) is crucial for distant metastasis of TNBC tumors. Thus, deciphering the latent mechanisms of LN metastasis is of great importance. A recent study illustrated that LN metastasis requires TNBC tumor cells to undergo a metabolic adaption toward FAO. The transcriptional coactivator YAP is a key molecule triggering the metabolic shift to FAO. A comparison of metabolomics and transcriptomics between primary and LN-metastatic tumors in mice found an upregulated FAO pathway in LN-metastatic tumor cells. FAO inhibition by etomoxir treatment significantly suppresses LN metastasis *in vivo*, implying that metabolic adaption to FAO is necessary for mediating LN metastasis. Therefore, blockage of FAO signaling or YAP expression may hamper LN metastasis in TNBC tumors (109).

Metabolic reprogramming is important for metastatic TNBC tumor cells as they adapt to the unique microenvironments at their secondary sites. However, unlike the traditional Warburg effect, TNBC metastatic tumor cells engage both glycolysis and OXPHOS (110). Interestingly, in TNBC-derived metastases to the lung, liver, and bone, TNBC cells display distinct metabolic profiles according to their site-selective metastatic potential. Specifically, liver metastases depend more on glycolysis, whereas metastases to bone or lung primarily rely on OXPHOS. This variation may result from interactions between metastatic TNBC cells and the microenvironments at the metastatic sites (111). Compared to lung or bone metastases, liver-metastatic breast cancer cells exhibit elevated HIF-1α/pyruvate dehydrogenase kinase 1 expression to promote the glycolytic phenotype, converting pyruvate into lactate rather than participating in mitochondrial oxidative metabolism, aiding their colonization and proliferation in the liver. One explanation for the diverging metabolic profile in liver-metastatic breast cancer cells may relate to the fact that the liver, as a gluconeogenic organ, can convert lactate to glucose and other metabolites. When transferred to cancer cells, these can be used for energy supply, giving the liver-specific metastases a distinct metabolic profile. On the other hand, bone- and lung-specific metastases divert pyruvate flux into mitochondrial oxidative metabolism to promote metastasis. Peroxisome proliferator-activated receptor-gamma coactivator 1 alpha (PGC-1α), the transcriptional co-activator of mitochondrial OXPHOS and biogenesis, is highly expressed in lung or bone metastases compared to those in the liver (111). PGC-1α enhances OXPHOS and mitochondrial function to foster the formation of secondary lung metastases and promote epithelial-to-mesenchymal processes rather than affect primary tumor growth (112). PGC-1α expression is elevated in lung metastases compared to primary tumors, indicating that metabolic pressures on the metastatic cancer cells contribute to upregulated PGC-1α expression. Comprehensive bioenergetic analyses further confirmed that PGC-1α expression augments the bioenergetic capacity of metastatic TNBC tumor cells to support their aggressiveness (112). The underlying mechanisms of these site-specific distinct metabolic phenotypes of TNBC metastases required detailed investigations in the future.

FAO is currently understood as a major metabolic program that provides a survival advantage to metastatic TNBC cells. Enhanced FAO sustains high mitochondrial energy production in metastatic TNBC tumor cells. Knockdown of CPT or etomoxir treatment in TNBC tumor cells confirmed the crucial role of FAO in driving TNBC-derived metastases (70). Alpha CUB-domain containing protein 1 (CDCP1) is a transmembrane glycoprotein, that drives TNBC cell metastasis. CDCP1 affects lipid metabolism by interacting with long-chain acyl-coenzyme A synthase 3, leading to reduced fatty acid activation and increased lipid utilization through FAO. Interestingly, this intensified FAO also facilitates OXPHOS to fuel TNBC metastasis. Blocking CDCP1 dimerization in TNBC tumor cells by expressing the released component of cleaved CDCP1 significantly hampers LD abundance and metastatic potential in TNBC cells. Thus, blocking this CDCP1-driven FAO and OXPHOS may inhibit TNBC metastasis (113). Aldo-keto reductase family 1 member B10 (AKR1B10) is another metastatic enhancer *in vivo*. AKR1B10 expression positively correlates with increased FAO pathway in TNBC. AKR1B10-high TNBC cells are characterized by impaired glycolytic phenotype and elevated levels of FAO. Elevated FAO limits oxidative stress toxicity, which benefits tumor cell survival in the pro-oxidative lung microenvironment. Etomoxir attenuates growth in AKR1B10-high tumor spheroids but not in the AKR1B10-high tumor spheroids. Thus, AKR1B10 may serve as a predictor of metastatic potential in TNBC and also as a target for future inhibitors in TNBC tumors (114). Another *in vivo* study reported impaired lung metastasis in myoferlin-deficient TNBC cells. More specifically, myoferlin depletion in TNBC tumor cells damages vesicle trafficking, leading to a misbalance between unsaturated and saturated fatty acids. An elevated ratio of unsaturated/saturated fatty acids in TNBC cells results in mitochondrial dysfunction and metabolic shift towards the glycolytic phenotype via activating AMPK signaling. Clinical statistics also reveal that TNBC patients harboring overexpressed-myoferlin tumors experience worse distant metastasis-free and overall survivals, implying the clinical significance of myoferlin in TNBC tumor metastasis (115). These observations illustrate that fatty acid metabolism has valuable implications for new therapeutic concepts and prognostic markers for the metastasis of TNBC tumors.

Proline catabolism catalyzed by proline dehydrogenase (PRODH) is also linked to TNBC-derived metastasis formation. In vivo studies revealed that PRODH is activated and proline accumulation is decreased in the lung metastatic foci compared to the primary foci, indicating a crucial role of PRODH in lung metastasis formation. Furthermore, PRODH inhibitors were effective for impairing lung metastasis formation without any impacts on the growth of primary tumors in orthotopic TNBC mouse models. Moreover, therapy with PRODH inhibitors does not lead to any damage in the adjacent normal tissue and organs *in vivo*, making PRODH a relatively safe druggable target (116). PRODH inhibition likely blocks micro-metastasis formation, since PRODH seems to be more essential at the beginning of the metastatic process. PRODH is thereby a druggable target to overcome TNBC metastasis.

## Impact of Metabolic Reprogramming on TNBC Chemoresistance

Cytotoxic chemotherapy is the backbone of systemic treatment for TNBC. Once TNBC patients develop resistance to chemotherapeutic agents, fewer treatment options remain. Thus, chemoresistance is another main cause of deaths for TNBC patients. Chemoresistant TNBC cells display an enhanced glycolytic phenotype, with increased glucose uptake and lactate fermentation (117). Silencing the key enzymes within the aerobic glycolysis pathway may enhance the anti-proliferation effect of chemotherapeutic agents. Increased of LDHA expression is found in paclitaxel-resistant TNBC cells, and LDHA knockdown or the LDH inhibitor oxamate both enables paclitaxel-resistant TNBC cells to be re-sensitized to paclitaxel. Intriguingly, the combined usage of paclitaxel and oxamate demonstrates a stronger killing effect on paclitaxel-resistant TNBC cells via cellular apoptosis compared to either paclitaxel or oxamate treatment alone (117). MYC and MCL1, which are frequently co-overexpressed in breast cancer stem cells (BCSC) of TNBC, act as enhancers of mitochondrial OXPHOS and synergistically upregulate HIF-1α expression. This enhanced mitochondrial OXPHOS further contributes to the enrichment of BCSC in TNBC, which induces the chemoresistance of TNBC cells. Moreover, HIF-1α inhibition attenuates the enrichment of BCSC and enables TNBC cells to regain some level of chemotherapeutic sensitivity (118).

Besides their role in glucose metabolism, fatty acids also play a role in mediating TNBC chemoresistance. FASN knockdown or inhibitors have roles in cisplatin-induced apoptosis. FASN inhibition attenuates cisplatin-induced apoptosis in receptor positive breast cancer cells, while enhancing cisplatin-induced apoptosis in TNBC tumor cells. This illustrates that FASN plays an oncogenic role in cisplatin-induced apoptosis of TNBC cells. FASN suppression in TNBC can therefore enhance cisplatin-induced apoptosis to overcome chemoresistance (119). Intensified FAO is also a characteristic of chemoresistant TNBC tumor cells. Notably, CPT1B has a negative relationship with chemotherapeutic sensitivity in breast cancer patients. Furthermore, the high expression of CPT1B driven by FAO in BCSC is essential for acquired paclitaxel resistance in TNBC tumor cells. JAK/STAT3/CPT1B-induced FAO promotes BCSC-associated chemoresistance in a series of TNBC cell lines. Treatments combining paclitaxel and the FAO inhibitor perhexiline are more effective against TNBC cells compared to either drug alone. This may result from FAO facilitating mitochondrial spare respiratory capacity, which constantly provides energy for breast cancer cells under conditions of chemotherapeutic stress. Thus, FAO inhibitors play a role in sensitizing chemoresistant TNBC tumor cells to chemotherapy (120).

Amino acid metabolism is also critical for TNBC chemotherapy. In chemoresistant TNBC cells, paclitaxel-based chemotherapy induces endoplasmic reticulum stress and promotes the interaction between ring finger protein 5 (RNF5) and SLC1A5. Consequently, SLC1A5 undergoes ubiquitination and degradation, leading to impaired glucose uptake, decreased mTOR activity and retarded TNBC tumor growth. Moreover, in TNBC cells, RNF5 inhibition also causes resistance to paclitaxel chemotherapy. High RNF5 expression and low SLC1A5 expression associate with positive prognosis in breast cancer. Thus, RNF5 and SLC1A5 status in TNBC patients may be valuable indicators of responsiveness to paclitaxel-based chemotherapy (121). PHGDH, in serine metabolism, is also involved in the sensitivity of TNBC tumor cells to chemotherapy. Both carboplatin and doxorubicin chemotherapy induce PHGDH-dependent BCSC enrichment. Treatment of PHGDH-deficient cells with carboplatin or doxorubicin at IC50, respectively, results in elevated mitochondrial ROS and enhanced apoptotic effects. Thus, PHGDH deficiency can attenuate chemotherapy-induced BCSC enrichment and sensitize breast cancer cells to chemotherapy (92).

## Conclusion

Our understanding of metabolic reprogramming in cancer cells has progressed greatly over the past decades. There is no doubt that an enhanced glycolytic phenotype supports the malignancy of TNBC tumors. Nevertheless, unlike the traditional Warburg effect that exhibits decreased activity of OXPHOS, dual alterations of OXPHOS have been reported in TNBC tumors, highlighting the need to identify the oncogenes driving the enhanced OXPHOS activity. Even though fatty acid synthesis and FAO are viewed as counterparts in the metabolic reprogramming of cancer cells, they may play synergistic roles in supporting the progression in TNBC cells. The glutamine-addicted phenotype of TNBC tumors makes glutamine-related enzymes potential therapeutic targets. Although serine and glycine are linked in their synthetic pathway, serine metabolism tends to be more significant for supporting the development of TNBC cells. The metabolic adaption of TNBC tumor cells also involves interplay with adjacent stromal cells since TNBC tumor cells need to survive and proliferate in a new microenvironment. Supportive effects from the surrounding stromal cells open new potential vulnerabilities of TNBC tumor cells. Metastasis and chemoresistance are currently the most severe challenges for TNBC patients. Therefore, further investigation is required into the process of how the metabolic reprogramming in TNBC tumor cells affects both of these processes.

## Author Contributions

XS: writing and elaborating the figures. MoW, MeW, XY, JG, TS, LY, XL, and HD: writing and reviewing the manuscript. YX: writing and reviewing the final version.

### Conflict of Interest

The authors declare that the research was conducted in the absence of any commercial or financial relationships that could be construed as a potential conflict of interest.
